# DATATOC: a novel conjugate for kit-type ^68^Ga labelling of TOC at ambient temperature

**DOI:** 10.1186/s41181-016-0007-3

**Published:** 2016-03-21

**Authors:** Johanna Seemann, Bradley Waldron, David Parker, Frank Roesch

**Affiliations:** 1 Institute of Nuclear Chemistry, Johannes Gutenberg University, Fritz-Strassmann-Weg 2, 55128 Mainz, Germany; 2 Department of Chemistry, Durham University, South Road, Durham, DH1 3LE UK

**Keywords:** ^68^Ga, DATA, TOC, Kit-type labelling

## Abstract

**Background:**

The widespread acceptance and application of ^68^Ga-PET depends on our ability to develop radiopharmaceuticals that can be prepared in a convenient and suitable manner. A kit-type labelling protocol provides such characteristics and requires chelators that can be radiolabelled under exceptionally mild conditions. Recently the DATA chelators have been introduced that fulfil these requirements. In continuing their development, the synthesis and radiolabelling of the first DATA bifunctional chelator (BFC) and peptide conjugate are described.

**Results:**

A BFC derived from the DATA ligand (2,2’-(6-((carboxymethyl)amino)-1,4-diazepane-1,4-diyl)diacetic acid) has been synthesised in five steps from simple building blocks, with an overall yield of 8 %. DATA^M5^-3^t^Bu (5-[1,4-Bis-tert-butoxycarbonylmethyl-6-(tert-butoxycarbonylmethyl-methyl-amino)-[1, 4]diazepan-6-yl]-pentanoic acid) has been coupled to [DPhe^1^][Tyr^3^]-octreotide (TOC) and the resulting peptide conjugate (DATATOC) radiolabelled with purified ^68^Ga derived *via* four different ^68^Ge/^68^Ga generator post-processing (PP) methods. The stability and lipophilicity of the radiotracer have been assessed and a kit-type formulation for radiolabelling evaluated. ^68^Ga-DATATOC has been prepared with a > 95 % radiochemical yield (RCY) within 1 (fractionated and acetone-PP) and 10 min (ethanol- and NaCl-PP) at 23 °C (pH 4.2–4.9, 13 nmol). The radiolabelled peptide is stable in the presence of human serum. Lipophilicity of ^68^Ga-DATATOC was calculated as logP = −3.2 ± 0.3, with a HPLC retention time (*t*_R_ = 10.4 min) similar to ^68^Ga-DOTATOC (logP = −2.9 ± 0.4, *t*_R_ = 10.3 min). Kit-type labelling from a lyophilised solid using acetone-PP based labelling achieves > 95 % RCY in 10 min at 23 °C.

**Conclusions:**

The favourable labelling properties of the DATA chelators have been retained for DATATOC. High radiochemical purity can be achieved at 23 °C in less than 1 min and from a kit formulation. The speed, reliability, ease, flexibility and simplicity with which ^68^Ga-DATATOC can be prepared makes it a very attractive alternative to current standards.

**Electronic supplementary material:**

The online version of this article (doi:10.1186/s41181-016-0007-3) contains supplementary material, which is available to authorized users.

## Background

The positron emitter ^68^Ga has a number of characteristics which make it a very attractive and promising radionuclide for PET imaging of disease and infection (Smith et al. 2013; Fani et al. 2008). In spite of this and numerous publications which provide support for its superiority, more established radionuclides and imaging modalities stand in its path (Buchmann et al. 2007; Tran et al. 2015). Interest in ^68^Ga-PET in Europe has grown over the last years, highlighted by the recent promotion of ^68^Ga -DOTATATE and -DOTATOC to orphan drug status in the United States (FDA Grants Orphan Drug Designation for ^68^Ga-DOTATOC. J Nucl Med 2014). ^99m^Tc became the work-horse of nuclear medicine in part due to the development of SPECT radiopharmaceuticals which can be prepared in a simple kit-type manner (Roesch 2013). The development of chelators that permit a similar protocol for ^68^Ga complement the inherent advantages of the ^68^Ge/^68^Ga-generator, paving the way for realisation of its full potential and delivery of advanced imaging diagnostics world-wide (Mukherjee et al. 2014; Velikyan et al. 2008).

The last decade of ^68^Ga-radiopharmaceutical chemistry has been dominated by studies with DOTATOC and its derivatives for the diagnosis of neuroendocrine tumours (NETs) (Frilling et al. 2010). The omnipresence of DOTA derivatives in ^68^Ga-PET arose from its success in other imaging applications (MRI and optical imaging with lanthanides) and its ability to provide an acceptable labelling profile and sufficient complex stability with ^68^Ga (Notni et al. 2011; Boros et al. 2010). Optimisation of radiolabelling protocols and the development of labelling modules have simplified radiopharmaceutical preparation, but the desire for further development remains (Ocak et al. 2010). The major inherent disadvantage of DOTA derivatives is the relatively harsh conditions required for radiolabelling (80–95 °C, 5–10 min, pH 3–4) that impose a number of limitations (Notni et al. 2011; Boros et al. 2010). Chelators which can be labelled quickly at room temperature would simplify labelling further and offer the potential for kit-type labelling akin to the prestigious ^99m^Tc kits. Temperature/pH sensitive targeting vectors (TVs) would benefit from these new chelators in particular, and widen the portfolio of ^68^Ga-based diagnostics. In recent times, there has been greater focus on the development of more efficient hexadentate chelators, and bifunctional derivatives of NOTA, TRAP, NOPO, DEDPA, CP256, HBED have been described that chelate ^68^Ga(III) rapidly at room temperature (Notni et al. 2011; Fani et al. 2012; Simeček et al. 2012; Boros et al. 2012; Berry et al. 2011; Eder et al. 2008).

**Fig. 1:**
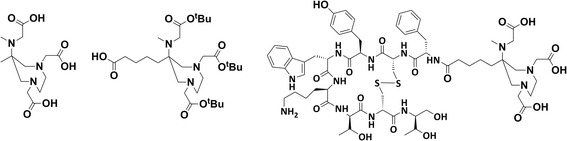
DATA^M^, bifunctional prochelator DATA^M5^-3^t^Bu and DATATOC (left to right)

The potential for kit-type labelling of biomolecule conjugates has been alluded to on numerous occasions, but a protocol which can achieve acceptable yields from a lyophilised solid at ambient temperature remains ‘the final frontier’ (Mukherjee et al. 2014; Velikyan et al. 2008 Asti et al. 2015; Waengler et al. 2011). The DATA chelators rapidly form stable complexes with ^68^Ga under exceptionally mild conditions befitting kit-type labelling (Waldron et al. 2013; Parker & Waldron 2013; Parker et al. 2013). The ligand DATA^M^ (Fig. 1) for instance, can enable radiochemical yields (RCYs) greater than 97 % at 23 °C in under 1 min (Seemann et al. 2015a). Earlier work showed that it is possible to label DATA chelators over the pH range 4–7, however for the purposes of this work it was decided that only optimum labelling conditions would be used in line with the desire to develop a room temperature kit-type labelling protocol (Waldron et al. 2013; Seemann et al. 2015a).

The main objective of this work has been to demonstrate the potential of a DATA conjugate towards development of kit-type labelling in a setting that holds considerable practical interest. The peptide TOC has been extensively studied with other chelators (DOTA, NODAGA, DTPA, DFO) in both imaging and therapeutic modalities, and has significant clinical and commercial importance (Lin et al. 2013; Eisenwiener et al. 2002; Dumont et al. 2011; Fani et al. 2011; Ugur et al. 2002). Hence, DATA conjugates were envisaged to allow comparative analysis.

The first step was to develop and synthesise a novel DATA bifunctional prochelator (DATA^M5^-3^t^Bu) that permits convenient amide conjugation to the N-terminus of TOC. A bifunctional derivative of DATA^M^ has been synthesised and conjugated to protected TOC using standard methods (Fig. 1). An initial radiolabelling evaluation of the conjugate (DATATOC, Fig. 1) has been carried out at ambient temperature, and a kit-type formulation tested.

## Methods

Reagents were purchased from Sigma-Aldrich® or Merck® and used without further purification. Purite® water used was filtered through a Millex® Millipore filter membrane (0.54 μm). Reaction progress was monitored using silica TLC-plates (silica 60 F_254_ 4.5 × 4.5 cm, Merck) and visualised with UV_254nm_ and/or KMnO_4_. Column chromatography was performed with silica gel 60 (Fisher Scientific®; 0.04–0.063 nm). NMR spectra (^1^H, ^13^C, HSQC, HMBC) were recorded on an Avance III HD 400 (Bruker, United States). Chemical shifts are given in ppm. MS (ESI) were performed with a Thermo Quest Navigator Instrument (Thermo Electron). Mass spec results are given as m/z in g/mol. HPLC was performed with a metal-free Dionex ICS-5000 system with a quaternary pump, an AS-50 auto sampler, UV/vis detector and automated fraction collector AFC-3000.

### Synthesis

**Fig. 2:**
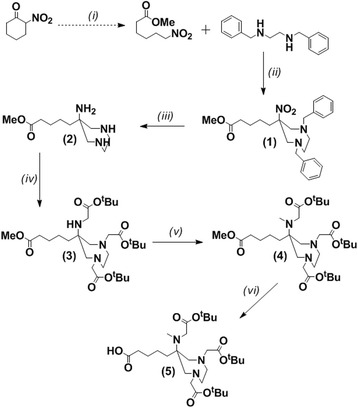
Synthesis of the unconjugated DATA prochelator - DATA^M5^-3^t^Bu. (i) Amberlyst-21, EtOH; (ii) CH_2_O, EtOH; (iii) CH_3_COOH, Pd(OH)_2_/C, H_2_, EtOH; (iv) BrCH_2_COO^t^Bu, K_2_CO_3_, MeCN; (v) CH_3_I, K_2_CO_3_, DCM : MeCN; (vi) LiOH, THF : H_2_O

The chemical identity of synthesised compounds has been confirmed by ^1^H-, ^13^C-NMR and HR MS with the exception of TOC conjugates, which have been characterised by HPLC and HRMS. ^1^H-, ^13^C-NMR and HRMS data for compounds prior to TOC conjugation are provided in the S.I. (Fig. 2).

*5-(1,4-Dibenzyl-6-nitro-[1, 4]diazepan-6-yl)-pentanoic acid methyl ester* (1) 2-Nitrocyclohexanone (0.608 g, 4.3 mmol) was added to Amberlyst A21 (1.216 g, 2 mass equivalents) in EtOH and stirred for 2 h at 60 °C under argon. N,N′-Dibenzyl-ethylenediamine (1.020 g, 4.3 mmol) and paraformaldehyde (0.446 g, 14.9 mmol) were added and the reaction stirred at 60 °C overnight. The mixture was filtered through Celite®, and solvent removed under reduced pressure. The resulting residue was re-dissolved in CHCl_3_ (40 mL) and washed successively with aqueous K_2_CO_3_ solution (2 × 30 mL, 0.1 M) and H_2_O (30 mL), dried over MgSO_4_, filtered and solvent removed under reduced pressure. Purification by silica gel column chromatography (DCM) afforded the title compound as a yellow oil (1.607 g, 85 %). R_f_ = 0.80 (DCM).

*5-(1,4-Dibenzyl-6-nitro-[1,4]diazepan-6-yl)-pentanoic acid methyl ester* (2) A catalytic amount of Pd(OH)_2_/C and acetic acid (50 μL, 0.87 mmol) was added to the protected triamine 1 (0.10 g, 0.29 mmol) in MeOH (20 mL), and the mixture agitated under an atmosphere of hydrogen for 3 h (1 atm H_2_). TLC (DCM) was used to confirm complete reduction of the nitro group and cleavage of the benzyl N-substituents. Pd(OH)_2_/C was removed using a Celite® filter. The solvent was removed under reduced pressure to afford a yellow oil. (0.065 g, 97 %)

*5-[1,4-Bis-tert-butoxycarbonylmethyl-6-(tert-butoxycarbonylmethyl-amino)-[1,4]diazepan-6-yl]-pentanoic acid methyl ester* (3) *tert*-Butyl-bromoacetate (0.567 g, 2.91 mmol) was added to 2 (0.208 g, 0.91 mmol) and K_2_CO_3_ (0.377 g, 2.73 mmol) in MeCN (25 mL), and the mixture stirred for 24 h at 368 K under an atmosphere of argon. The reaction was monitored by TLC (hexane/ethyl acetate; 1:1) for formation of the tetra-alkylated derivative. The solvent was removed under reduced pressure, and the resulting oil re-dissolved in CHCl_3_ (25 mL) and washed successively with aqueous K_2_CO_3_ solution (2 × 25 mL, 0.1 M) and H_2_O (25 mL), dried over MgSO_4_, filtered and solvent removed under reduced pressure. Purification by silica gel column chromatography (hexane/ethyl acetate, 2:1 → 1:1) afforded a yellow oil (0.229 g, 44 %). R_f_ = 0.35 (hexane/ethyl acetate; 2:1).

*5-[1,4-Bis-tert-butoxycarbonylmethyl-6-(tert-butoxycarbonylmethyl-methyl-amino)-[1,4]diazepan-6-yl]-pentanoic acid methyl ester* (4) Iodomethane (0.023 g, 0.16 mmol) was added to 3 (0.104 g, 0.18 mmol) and K_2_CO_3_ (0.025 g, 0.18 mmol) in DCM/MeCN (3:1) cooled in an ice-bath. The reaction mixture was allowed to warm to room temperature and left overnight. The solvent was removed under reduced pressure and the resulting oil re-dissolved in CHCl_3_ (20 mL), filtered, and washed successively with aqueous K_2_CO_3_ solution (2 × 20 mL, 0.1 M) and H_2_O (20 mL), dried over MgSO_4_, filtered and solvent removed under reduced pressure. Purification by silica gel column chromatography (hexane/ethyl acetate, 3:1 → 2:1) afforded a yellow oil (0.043 g, 46 %). R_f_ = 0.38 (hexane/ethyl acetate; 2:1).

*5-[1,4-Bis-tert-butoxycarbonylmethyl-6-(tert-butoxycarbonylmethyl-methyl-amino)-[1,4]diazepan-6-yl]-pentanoic acid* (5) LiOH (0.009 g, 0.039 mmol) dissolved in H_2_O (0.5 mL) was added to 4 (0.010 g, 0.023 mmol) in THF (0.5 mL), and the mixture stirred at 298 K. The reaction was monitored using LC-ESI MS for ester cleavage. Once complete, the solvent was removed by lyophilisation. H_2_O (5 mL) was added and removed by lyophilisation, and the procedure repeated two more times. The resulting solid was washed with ice-cold DCM (0.5 mL), and dried *in vacuo* to afford a waxy yellow solid (0.009 g, 70 %).

### Synthesis of DATATOC

Commercially available protected TOC [(D)Phe^1^,Tyr(^t^Bu)^3^,D-Trp(Boc)^4^,Lys(Boc)^5^, Thr(^t^Bu)^6,8^]-octreotide was coupled to 5 using N,N-diisopropylethylamine (DIPEA) and O-(benzotriazol-1-yl)-N,N,N′,N′-tetramethyluronium hexafluorophosphate (HBTU). The protected TOC (0.024 g, 0.017 mmol), HBTU (0.0010 g, 0.029 mmol), DIPEA (0.0016 g, 0.12 mmol) and 5 (0.015 g, 0.026 mmol) were added to dry DMSO (2 mL) and stirred at ambient temperature for 4 h. The solvent was removed under reduced pressure, and the resulting residue dissolved in 1 mL MeCN. The precipitate was removed by centrifugation, and solvent removed by lyophilisation. The resulting oil was purified by HPLC (C_18_-RP; A = 0.1 % TFA in H_2_O; B = MeCN; 0–2 min 35 % A; linear gradient to 10 % A at 14 min, 14–24 min 10 % A). The fully protected product (6) eluted with a retention time of 21 min (0.014 g, 41 %). MS ES^+^ (m/z) found: 1957.1133 [M + H]^+^; C_100_H_158_N_13_O_22_S_2_ calcd for: 1957.1086.

**Fig. 3:**
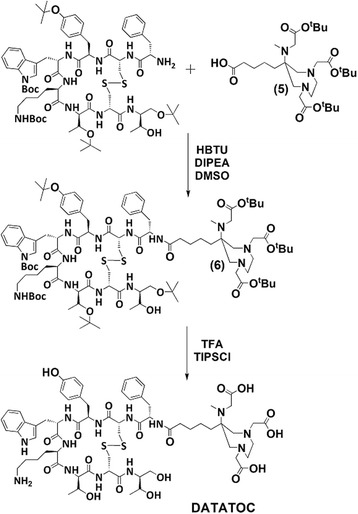
Synthesis of DATATOC

Deprotecting of the chelator and TOC was performed by dissolving 6 (0.050 g, 2.6 × 10^−5^ mmol) in a mixture of TFA/water/triisopropylsilane (95:2.5:2.5) and stirring at ambient temperature overnight. Once complete, the solvent was removed by lyophilisation. H_2_O/MeCN (1 mL, 1:1) was added and removed by lyophilisation, and the procedure repeated two more times. Purification was performed by preparative HPLC (C_18_-RP; A = 0.1 % TFA in water and B = MeCN; linear gradient: 0 min 80 % A, 15 min 65 % A, t_R_ (product) = 12 min). DATATOC was eluted with a retention time of 12 min (0.006 g, 76 %). HR MS ES^+^ (m/z) found: 1421.6259 [M + H]^+^; calcd. for C_66_H_94_N_13_O_18_S_2_: 1420.6281 (Fig. 3).

### Radiochemistry

All radiochemical evaluations were conducted using chemicals of the highest available purity grade. Volumes were measured using an Eppendorf pipette. A TiO_2_-based ^68^Ge/^68^Ga generator (Cyclotron Co., Obninsk, Russia) was used for all radiochemical evaluations. Four post-processing (PP) methods were used to purify and pre-concentrate the radioactive eluate: fractionation, acetone-, ethanol- and NaCl-based. Procedures were carried out, and solutions required prepared, as detailed in the relevant publication (Zhernosekov et al. 2007; Mueller et al. 2012; Eppard et al. 2014; Breeman et al. 2005). pH was measured using a Mettler-Toledo, SevenEasy pH. Radio-TLC was performed using silica-60 TLC plates (Merck F254, 4.5 × 4.5 cm), and eluted using 0.1 M citrate buffer (pH 4). Eluted radio-TLC plates were analysed using a flat-bed imaging scanner (Instant Imager, Canberra Packard).

### Radiolabelling

Experiments were carried out in triplicate, and the amount of DATATOC used was constant in each case (13 nmol) taken from a 1 mg/mL stock solution. The volume of eluate differed according to the post-processing used, but was diluted as necessary so that ~ 100 MBq ^68^Ga was used for radiolabelling in each instance. Radiolabelling experiments were maintained at 23 °C by means of a heater-shaker device (DITABIS MHR 11), which was also used to agitate (400 rpm) radiolabelling solutions. TLC samples (1 μL) were taken at 1, 3, 5 and 10 min. The optimised labelling conditions vary according to the PP method applied. The important differences and approach used in each case are summarised in Table 1.

**Table 1:** Key features of the radiolabelling procedures used

PP method	Eluate volume (mL)	Labelling media	Buffer volume (mL)	DATATOC (nmol/μM)	Labelling pH
Fractionation	0.900	1.00 M NH_4_OAc	0.200	13 / 11.8	4.9
Acetone	0.400	0.20 M NaOAc	1.000	13 / 9.29	4.5
Ethanol	1.000	1.00 M NH_4_OAc	1.500	13 / 5.20	4.9
NaCl	0.510	1.00 M NH_4_OAc	3.500	13 / 3.24	4.2

### Stability of ^68^Ga-DATATOC

Stability was assessed following formation of ^68^Ga-DATATOC using ethanol-PP ^68^Ga and performed in triplicate. The stability was assessed by incubating ^68^Ga-DATATOC (50 μL) in an excess of human serum (300 μL) at 37 °C and pH 7 (1.0 M phosphate buffered saline) for 2 h. Samples (1 μL) were taken at 30, 60, 90 and 120 min and analysed by radio-TLC.

### Lipophilicity of ^68^Ga-DATATOC

The lipophilicity was determined using the shake-flask method (logP) and qualitatively by radio-HPLC relative to ^68^Ga-DOTATOC (Notni et al. 2013; Du et al. 1998). Radiolabels were prepared following acetone-PP. Analytical radio-HPLC (LiChrospher 100-RP-18EC, 1 mL/min) for lipophilicity studies was based on a gradient using H_2_O (A) and MeCN (B), each containing 0.1 % TFA. Mobile phase gradient: 0–1.5 min 1 % B, 1.5–10.5 min 99 % B, 11.5 min 99 % B, 12–15 min 1 % B. The HPLC was coupled to a UV (Hitachi L-7400) and a radioactivity (Gamma Raytest) detection system. HPLC grade solvents were used and degassed by sonication for 15–20 min prior to use.

### Kit-type labelling

Formulations for labelling following fractionation and acetone-PP were used to investigate the possibility of kit-type labelling.

*Acetone-PP:* The precursor solution was prepared as per Table 1 and lyophilised into a vial suitable for labelling. The post-processed ^68^Ga was diluted to the labelling volume (1.4 mL) using H_2_O and added to the lyophilised solid. The mixture was agitated on a test-tube vortex for 2 s and left to stand at ambient temperature.

*Fractionated* ^*68*^ *Ga:* owing to the nature of the labelling media used, it was not possible to prepare a lyophilised solid. After addition of ^68^Ga, the labelling solution was agitated on a test-tube vortex for 2 s and left to stand at ambient temperature. After 10 min a 1 μL sample was extracted and analysed by radio-TLC.

## Results

### Cold-Synthesis

The synthesis of DATA^M5^-3^t^Bu was successfully carried out in five-steps in an 8 % yield. A further two steps were required to afford DATATOC in a yield of 31 %.

### Radiochemical evaluations

**Fig. 4:**
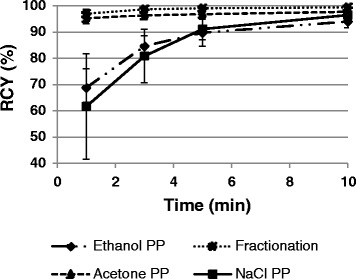
Time dependent labelling for the formation of ^68^Ga-DATATOC using ^68^Ga post-processed by fractionation, acetone-, ethanol- and NaCl-based procedures

The optimised labelling of DATATOC using ^68^Ga PP by four common procedures is shown in Fig. 4. The labelling volume varies depending on the PP method used, and as a result the precursor concentration is not the same in each case. Radiochemical yields (RCYs given with ± SD, n = 3) greater than 95 % were achieved with each type of PP eluate within 10 min, and from a kit formulation. Radio-HPLC confirmed that the radiolabelled product was the same in each instance.

In human serum 98.7 % of the initially present ^68^Ga-DATATOC remained intact after 2 h. The lipophilicity of ^68^Ga-DATATOC and ^68^Ga-DOTATOC, determined using the shake-flask method, are logP = −3.22 ± 0.32 and −2.93 ± 0.37 respectively. The respective retention times from radio-HPLC are 10.4 and 10.3 min.

## Discussion

The synthesis of DATA^M5^-3^t^Bu followed the same synthetic pathway as that of DATA^M^, with the only modification being the type of reagent used in the first step. A similar synthetic route was reported for an AAZTA conjugate with the same linker functionality, and the product labelled with ^68^Ga (Manzoni et al. 2012). In our experience, the heptadentate AAZTA ligand itself is unsuitable for chelation of ^68^Ga due to the formation of multiple radiolabelled species (Baranyai et al. 2009). The spacer and linker moieties for conjugation are incorporated in the first step during formation of the 7-membered diazepine ring. A five-carbon spacer, attached to the quaternary carbon of the ring, possesses an acid which is orthogonally protected as an ester relative to the chelating acids, to ensure selective conjugation of TOC. The key step is the N-methylation (step 4), necessary to prevent an internal cyclisation with an adjacent acetate, where care is required to prevent formation of a quaternary amine. A suitably protected TOC derivative was conjugated in solution, with subsequent deprotecting of the chelator and peptide occurring simultaneously.

There are four commonly used ^68^Ga generators and four main PP methods (acetone-, ethanol-, NaCl- based and fractionation) (Zhernosekov et al. 2007; Mueller et al. 2012; Eppard et al. 2014; Breeman et al. 2005). Each generator and PP method has particular advantages and the combination used varies from group to group. This variety adds a layer of complication because the optimum labelling conditions and labelling efficiency of a given precursor can vary depending on the combination applied (Seemann et al. 2015a). A precursor which can be labelled reliably using any combination of the available technology is desirable, and may facilitate easier translation of labelling between institutions. The labelling experiments have been performed using an Obninsk (EZAG) generator eluted with 0.1 M HCl. The setup is intended to serve as an example to demonstrate the versatility of labelling procedures which can be applied to DATATOC. Further validation of any kit-type labelling protocol requires implementation with a market authorised ^68^Ga-generator. Radiolabelling of DATATOC was evaluated at ambient temperature using ^68^Ga PP by each of the four methods, optimised in terms of the labelling pH. Remarkably, RCYs > 95 % were achieved in only 1 min with fractionated and acetone-PP ^68^Ga, and > 98 % in 10 min regardless of the PP method applied. The precursor concentration increases along the series NaCl-, ethanol-, acetone- and fractionation PP, and is most likely the reason for the increasing rate of labelling along the same series. According to European Pharmacopeia governing ^68^Ga-DOTATOC a radiochemical purity of at least 91 % is required (measured by HPLC and TLC) prior to in vivo administration, which may require the inclusion of a post-labelling purification (Virgolini et al. 2010). Based on the results gathered such a procedure to improve the RCY is redundant for the preparation of ^68^Ga-DATATOC. This is analogous to the very successful ^99m^Tc-kits and not only simplifies the process but also saves time – a valuable commodity considering the short half-life of ^68^Ga compared to ^99m^Tc and ^18^F.

The influence of lower precursor concentrations has not been tested, but based on the radiolabelling profile it is apparent that there is scope for reduction. Nevertheless, the concentration used compares well with other ^68^Ga-labelled peptide preparations (Notni et al. 2011; Berry et al. 2011; Lin et al. 2013; Eisenwiener et al. 2002; Dumont et al. 2011; Fani et al. 2011; Ugur et al. 2002; Virgolini et al. 2010; Wester et al. 1997). Based on the results it is evident that the favourable properties of the original DATA^M^ chelator have not been negatively affected to any significant extent by conjugation to TOC. Surprisingly, with NaCl- and ethanol-PP ^68^Ga, DATATOC shows superior labelling kinetics to the unfunctionalised chelator – DATA^M^ (Seemann et al. 2015b).

There is no evidence for radiolysis of ^68^Ga-DATATOC on radio-HPLC following labelling. In human serum there was no appreciable release of ^68^Ga over 2 h, indicating a high metabolic and kinetic stability (98.7 %).

The relative lipophilicities ^68^Ga-DATATOC and ^68^Ga-DOTATOC determined by radio-HPLC and shake-flask methods are consistent and show that the former is marginally more lipophilic. Publications involving ^68^Ga-labelled TOC with different chelators have highlighted the importance of the chelator in terms of *in vivo* performance, suggesting that the BFC could be tailored to the TV used (Lin et al. 2013; Eisenwiener et al. 2002; Dumont et al. 2011; Fani et al. 2011; Ugur et al. 2002; Wester et al. 1997). Previous work has shown that is possible to modify the lipophilicity of DATA chelators without disturbing the radiolabelling characteristics, offering the potential to look at this relationship more closely (Seemann et al. 2015b).

The feasibility of a kit-type formulation has been assessed using fractionated and acetone-PP ^68^Ga and produced excellent results. Virtually quantitative yields were obtained in less than 10 min (shorter intervals not analysed) from a lyophilised solid formulation with acetone-PP ^68^Ga. A similar result was achieved with fractionated ^68^Ga (97 % RCY). However, it was not possible to lyophilise the precursor formulation due to the tendency of ammonia acetate to sublime under the reduced pressure required to lyophilise the labelling media. This issue can be avoided if the ammonium acetate required for labelling is incorporated into the water used to dilute activity prior to labelling rather than as part of the lyophilised solid.

To the best of our knowledge, this is the first example of a ^68^Ga- radiopharmaceutical that can be prepared from a lyophilised solid at ambient temperature in less than 10 min. There are examples of ^68^Ga kits requiring elevated temperatures, but we are only aware of a single ambient temperature kit where labelling of a NOTA-peptide conjugate occurred from a pre-dissolved formulation (Mukherjee et al. 2014; Velikyan et al. 2008). Beyond the development of kit-type formulations, these favourable characteristics can also be exploited through the development of previously inaccessible temperature sensitive biomolecules.

It could be argued that the PP procedures currently available do not compliment the advantages of kit-type labelling because they are manual procedures with a sequence of steps. At this stage this part of the manufacturing process then does not correspond to a kit-type preparation. However, the PP protocols are still simple and routine such that there remains substantial benefits to a very simple, fast and reliable labelling method. Future improvements to the generator and elution procedures may lend themselves better to kit-type labelling, but in the meantime there are sophisticated modules capable of post-processing the eluate with minimal input from the user which would benefit from kit-type labelling.

## Conclusion

The first DATA bifunctional chelator has been synthesised and conjugated to TOC in a short seven-step synthesis using affordable starting materials. DATATOC displays remarkable radiolabelling characteristics with > 95 % RCYs possible within 1 min at ambient temperature (pH 4.9, 13 nmol) providing a radiotracer with high human serum stability. ^68^Ga-DATATOC can be efficiently prepared using ^68^Ga post-processed by the full range of commonly used methods. The labelling protocols are facile, reliable, robust and do not require non-standard equipment and reagents. Initial efforts towards the development of a kit-type formulation analogous to ^99m^Tc have been successful, and are now being shared with other research groups to assess performance in different settings. *In vivo* and *in vitro* studies comparing ^68^Ga-labelled DATATOC, DOTATOC and NODAGATOC are underway.

^68^Ga-DATA conjugated radiopharmaceuticals may meet the five main definitions of kit-like preparations: A radiopharmaceutical which can be prepared in a (i) sufficient radiochemical yield (ii) from a lyophilised solid (iii) at room temperature (iv) within a short time (v) that does not require post-labelling purification to meet pharmacopeia standards. The radiochemical performance of DATATOC highlights the potential of DATA-conjugates to carry ^68^Ga-PET into widespread application through the availability of a true ‘kit-type’ formulation, a facile and cost effective synthesis as well as the preparation of previously inaccessible radiotracers.

### Ethical approval

This article does not contain any studies with human participants or animals performed by any of the authors.

## Additional file

Additional file 1: Figure S1. RadioTLC for kit-type ^68^Ga-labelling of DATATOC at 1, 3, 5, 10 and 15 min. **Figure S2**: Radioactive (gamma) and UV traces for the kit-type labelling of DATATOC with ^68^Ga. The unlabelled ligand is evident at 9.1 min (confirmed by injection of the free ligand only) and the ^68^Ga-labelled complex at 13.0 min. **Figure S3**: Illustrative example of RadioTLC for stability study of ^68^Ga-DATATOC 30, 60, 90 and 120 min after exposure to human serum. (DOCX 107 kb)

## References

[CR1] Asti M, Iori M, Capponi PC, Rubagotti S, Fraternali A, Versari A (2015). Development of a simple kit-based method for preparation of pharmaceutical-grade ^68^Ga-DOTATOC. Nucl Med Commun..

[CR2] Baranyai Z, Uggeri F, Giovenzana GB, Bényei A, Bruecher E, Aime S (2009). Equilibrium and kinetic properties of the lanthanoids(III) and various divalent metal complexes of the heptadentate ligand AAZTA. Chem Eur J.

[CR3] Berry DJ, Ma Y, Ballinger JR, Tavaré R, Koers A, Sunassee K (2011). Efficient bifunctional ^68^Ga chelators for positron emission tomography: tris(hydroxypyridinone) ligands. Chem Commun.

[CR4] Boros E, Ferreira CL, Cawthray JF, Price EW, Patrick BO, Wester DW (2010). Acyclic chelate with ideal properties for ^68^Ga PET imaging agent elaboration. J Am Chem Soc.

[CR5] Boros E, Ferreira CL, Yapp DTT, Gill RK, Price EW, Adam MJ (2012). RGD conjugates of the H_2_dedpa scaffold: synthesis, labeling and imaging with ^68^Ga. Nucl Med Biol.

[CR6] Breeman WAP, Jong M, Blois E, Bernard BF, Konijnenberg M, Krenning EP (2005). Radiolabelling DOTA-peptides with ^68^Ga. Eur J Nucl Med Mol Imaging.

[CR7] Buchmann I, Henze M, Engelbrecht S, Eisenhut M, Runz A, Schaefer M (2007). Comparison of ^68^Ga-DOTATOC PET and ^111^In-DTPAOC (Octreoscan) SPECT in patients with neuroendocrine tumours. Eur J Nucl Med Mol Imaging.

[CR8] Du CM, Valko K, Bevan C, Reynolds D, Abraham MH (1998). Rapid Gradient RP-HPLC Method for Lipophilicity Determination: A Solvation Equation Based Comparison with Isocratic Methods. Anal Chem..

[CR9] Dumont RA, Deininger F, Haubner R, Maecke HR, Weber WA, Fani M (2011). Novel ^64^Cu- and ^68^Ga-labeled RGD conjugates show improved PET imaging of α_ν_β_3_ integrin expression and facile radiosynthesis. J Nucl Med.

[CR10] Eder M, Waengler B, Knackmuss S, LeGall F, Little M, Haberkorn U (2008). Tetrafluorophenolate of HBED-CC: a versatile conjugation agent for ^68^Ga-labeled small recombinant antibodies. Eur J Nucl Med Mol Imaging.

[CR11] Eisenwiener K, Prata MIM, Buschmann I, Zhang H, Santos AC, Wenger S (2002). NODAGATOC, a New Chelator-Coupled Somatostatin Analogue Labeled with ^67/68^Ga and ^111^In for SPECT, PET, and Targeted Therapeutic Applications of Somatostatin Receptor (hsst2) Expressing Tumors. Bioconjugate Chem.

[CR12] Eppard E, Wuttke M, Nicodemus PL, Roesch F (2014). Ethanol-Based Post-processing of Generator-Derived ^68^Ga Toward Kit-Type Preparation of ^68^Ga-Radiopharmaceuticals. J Nucl Med.

[CR13] Fani M, André JP, Maecke HR (2008). ^68^Ga-PET: a powerful generator-based alternative to cyclotron-based PET radiopharmaceuticals. Contrast Media Mol Imaging.

[CR14] Fani M, Del Pozzo L, Abiraj K, Mansi R, Tamma ML, Cescato R (2011). PET of somatostatin receptor-positive tumors using ^64^Cu- and ^68^Ga-somatostatin antagonists: the chelate makes the difference. J Nucl Med.

[CR15] Fani M, Tamma M, Nicolas GP, Lasri E, Medina C, Raynal I (2012). In vivo imaging of folate receptor positive tumor xenografts using novel ^68^Ga-NODAGA-folate conjugates. Mol Pharm.

[CR16] FDA Grants Orphan Drug Designation for ^68^Ga-DOTATOC. J Nucl Med. 2014;55(1):10 N.

[CR17] Frilling A, Sotiropoulos GC, Radtke A, Malago M, Bockisch A, Kuehl H (2010). The impact of ^68^Ga-DOTATOC positron emission tomography/computed tomography on the multimodal management of patients with neuroendocrine tumors. Ann Surg.

[CR18] Lin M, Welch MJ, Lapi SE (2013). Effects of chelator modifications on ^68^Ga-labeled [Tyr ^3^]octreotide conjugates. Mol Imaging Biol.

[CR19] Manzoni L, Belvisi L, Arosio D, Bartolomeo MP, Bianchi A, Brioschi C (2012). Synthesis of Gd and ^68^Ga complexes in conjugation with a conformationally optimized RGD sequence as potential MRI and PET tumor-imaging probes. ChemMedChem.

[CR20] Mueller D, Klette I, Baum RP, Gottschaldt M, Schultz MK, Breeman WAP (2012). Simplified NaCl based ^68^Ga concentration and labeling procedure for rapid synthesis of ^68^Ga radiopharmaceuticals in high radiochemical purity. Bioconjugate Chem.

[CR21] Mukherjee A, Pandey U, Chakravarty R, Sarma HD, Dash A (2014). Single vial kit formulation for preparation of PET radiopharmaceutical: ^68^Ga-DOTA-TOC. J Radioanal Nucl Chem.

[CR22] Notni J, Šimeček J, Hermann P, Wester H (2011). TRAP, a powerful and versatile framework for ^68^Ga radiopharmaceuticals. Chem Eur J.

[CR23] Notni J, Pohle K, Wester H (2013). Be spoilt for choice with radiolabelled RGD peptides: preclinical evaluation of ^68^Ga-TRAP(RGD)_3_. Nucl Med Biol.

[CR24] Ocak M, Antretter M, Knopp R, Kunkel F, Petrik M, Bergisadi N (2010). Full automation of ^68^Ga labelling of DOTA-peptides including cation exchange prepurification. Appl Radiat Isot.

[CR25] Parker D, Waldron BP (2013). Conformational analysis and synthetic approaches to polydentate perhydro-diazepine ligands for the complexation of gallium(III). Org Biomol Chem.

[CR26] Parker D, Waldron BP, Yufit DS (2013). Crystallographic and solution NMR structural analyses of four hexacoordinated gallium(III) complexes based on ligands derived from 6-amino-perhydro-1,4-diazepine. Dalton Trans.

[CR27] Roesch F (2013). Past, present and future of ^68^Ge/^68^Ga generators. Appl Radiat Isot..

[CR28] Seemann J, Eppard E, Waldron BP, Ross TL, Roesch F (2015). Cation exchange-based post-processing of ^68^Ga-eluate: A comparison of three solvent systems for labelling of DOTATOC, NO2AP^BP^ and DATA^M^. Appl Radiat Isot..

[CR29] Seemann J, Waldron BP, Roesch F, Parker D (2015). Approaching ‘kit-type’ labelling with ^68^Ga: the DATA chelators. Chem Med Chem.

[CR30] Simeček J, Zemek O, Hermann P, Wester H, Notni J (2012). A monoreactive bifunctional triazacyclononane phosphinate chelator with high selectivity for ^68^Ga. ChemMedChem.

[CR31] Smith DL, Breeman WAP, Sims-Mourtada J (2013). The untapped potential of ^68^Gallium-PET: the next wave of ^68^Ga-agents. Appl Radiat Isot.

[CR32] Tran K, Khan S, Taghizadehasl M, Palazzo F, Frilling A, Todd JF (2015). ^68^Ga dotatate PET/CT is superior to other imaging modalities in the detection of medullary carcinoma of the thyroid in the presence of high serum calcitonin. Hell J Nucl Med..

[CR33] Ugur O, Kothari PJ, Finn RD, Zanzonico P, Ruan S, Guenther I (2002). ^66^Ga labeled somatostatin analogue DOTA-DPhe-Tyr-octreotide as a potential agent for positron emission tomography imaging and receptor mediated internal radiotherapy of somatostatin receptor positive tumors. Nucl Med Biol..

[CR34] Velikyan I, Maecke H, Langstrom B (2008). Convenient preparation of ^68^Ga-based PET-radiopharmaceuticals at room temperature. Bioconjug. Chem..

[CR35] Virgolini I, Ambrosini V, Bomanji JB, Baum RP, Fanti S, Gabriel M (2010). Procedure guidelines for PET/CT tumour imaging with ^68^Ga-DOTA-conjugated peptides: ^68^Ga-DOTA-TOC, ^68^Ga-DOTA-NOC, ^68^Ga-DOTA-TATE. Eur J Nucl Med Mol Imaging.

[CR36] Waengler C, Waengler B, Lehner S, Elsner A, Todica A, Bartenstein P (2011). A universally applicable ^68^Ga-labeling technique for proteins. J Nucl Med.

[CR37] Waldron BP, Parker D, Burchardt C, Yufit DS, Zimny M, Roesch F (2013). Structure and stability of hexadentate complexes of ligands based on AAZTA for efficient PET labelling with ^68^Ga. Chem Commun.

[CR38] Wester H, Brockmann J, Roesch F, Wutz W, Herzog H, Smith-Jones P (1997). PET-pharmacokinetics of ^18^F-octreotide: A comparison with ^67^Ga-DFO and ^86^Y-DTPA-octreotide. Nucl Med Biol.

[CR39] Zhernosekov KP, Filosofov DV, Baum RP, Aschoff P, Bihl H, Razbash AA (2007). Processing of generator-produced ^68^Ga for medical application. J Nucl Med.

