# Supplementary material for: DATATOC: a novel conjugate for kit-type 68Ga labelling of TOC at ambient temperature
Source: EJNMMI Radiopharm Chem. 2016 Mar 21;1:4. doi: 10.1186/s41181-016-0007-3 (PMC5843802; doi:10.1186/s41181-016-0007-3)
Supplement: Supplementary file 1 — RadioTLC for kit-type 68Ga-labelling of DATATOC at 1, 3, 5, 10 and 15 min. Figure S2: Radioactive (gamma) and UV traces for the kit-type labelling of DATATOC with 68Ga. The unlabelled ligand is evident at 9.1 min (confirmed by injection of the free ligand only) and the 68Ga-labelled complex at 13.0 min. Figure S3: Illustrative example of RadioTLC for stability study of 68Ga-DATATOC 30, 60, 90 and 120 min after exposure to human serum. (DOCX 107 kb) [file 41181_2016_7_MOESM1_ESM.docx]

**Supplementary Information**

Johanna Seemann, David Parker, Frank Roesch, Bradley P. Waldron.

^1^H-, ^13^C- NMR and HR MS characterisation of synthesised compounds.

*5-(1,4-Dibenzyl-6-nitro-[1,4]diazepan-6-yl)-pentanoic acid methyl ester* **(1)**. ^1^H NMR (CDCl_3_): δ 0.78 (2H, m, C***H_2_***CH_2_COO); 1.32 (2H, m, C***H_2_***(CH_2_)_2_COO); 1.59 (2H, m, C***H_2_***CNO_2_); 2.12 (2H, m, C***H_2_***COO); 2.63 (4H, m, NC***H_2_***C***H_2_***N); 2.97/3.53 (4H, dd, J 14 NC***H_2_***CNO_2_); 3.59/3.75 (4H, dd, J 14, NC***H_2_***Ph); 3.66 (3H, s, OC***H_3_***); 7.29 (10H, m, CH_2_***Ph***). ^13^C NMR (CDCl_3_): δ 22.6 (***C***H_2_CH_2_COO); 24.6 (***C***H_2_(CH_2_)_2_COO); 33.6 (***C***H_2_CNO_2_); 36.4 (***C***H_2_COO) 51.5 (O***C***H_3_); 58.9 (N***C***H_2_***C***H_2_N); 61.8 (N***C***H_2_CNO_2_); 64.9 (***C***H_2_Ph); 94.81 (***C***NO_2_); 127.3/128.3/129.1 (***C***H); 139.1 (***C***_quat_(Ph)); 173.6 (***C***OO). MS ES+ (m/z): found 440.2566 [M + H]^+^; C_25_H_34_N_3_O_4_ calcd for: 440.2549.

*5-(1,4-Dibenzyl-6-nitro-[1,4]diazepan-6-yl)-pentanoic acid methyl ester* **(2)**. ^1^H NMR (CDCl_3_): δ 1.39 (2H, m, C***H_2_***CNH_2_); 1.58 (4H, m, C***H_2_***C***H_2_***CH_2_COO); 2.32 (2H, t, J 7, C***H_2_***COO); 2.91-3.32 (8H, dd + m, NC***H_2_***C***H_2_***N & NC***H_2_***CNH_2_); 3.66 (3H, s, OC***H_3_***). ^13^C NMR (CDCl_3_): δ 22.2 (***C***H_2_CH_2_CNH_2_); 22.8 (***C***H_2_CH_2_COO); 25.1 (***C***H_2_CNH_2_); 33.5 (***C***H_2_COO); 36.7 (***C***NH2); 47.5 (N***C***H_2_***C***H_2_N); 51.5 (O***C***H_3_); 55.0 (N***C***H_2_CNH_2_); 173.8 (***C***OO). MS ES+ (m/z): found 230.1867 [M + H]^+^; C_11_H_24_N_3_O_2_ calcd for 230.1868.

*5-[1,4-Bis-tert-butoxycarbonylmethyl-6-(tert-butoxycarbonylmethyl-amino)-[1,4]diazepan-6-yl]-pentanoic acid methyl ester* **(3)**. ^1^H NMR (CDCl_3_): δ 1.32 (4H, m, C***H_2_***C***H_2_***CH_2_COO); 1.48 (27H, br s, ***^t^Bu***); 1.61 (2H, m, C***H_2_***CNH); 2.32 (2H, t, J 7, C***H_2_***COOCH_3_); 2.70 (4H, dd, J 14, NC***H_2_***CN); 2.80 (4H, m, NC***H_2_***C***H_2_***N); 3.24 (2H, s, NHC***H_2_***COO); 3.31 (4H, s, NC***H_2_***COO), 3.68 (3H, s, OC***H_3_***). ^13^C NMR (CDCl_3_): δ 22.6 (***C***H_2_CH_2_COO); 25.6 (***C***H_2_CN); 28.1 (C(***C***H_3_)_3_); 34.0 (***C***H_2_COO); 51.4 (O***C***H_3_); 57.3 (N***C***H_2_***C***H_2_N); 57.9 (***C***NH) 61.9 (N***C***H_2_COO); 63.4 (N***C***H_2_CN); 80.8 (***C***(CH_3_)_3_); 171.0 (NCH_2_***C***OO); 171.8 (NHCH_2_***C***OO); 174.1 (***C***OOCH_3_) . MS ES+ (m/z): found 572.3901 [M + H]^+^; calcd for C_29_H_54_N_3_O_8_: 572.3910.

*5-[1,4-Bis-tert-butoxycarbonylmethyl-6-(tert-butoxycarbonylmethyl-methyl-amino)-[1,4]diazepan-6-yl]-pentanoic acid methyl ester* **(4)**. ^1^H NMR (CDCl_3_): δ 1.38 (2H, m, C***H_2_***CH_2_C); 1.48 (27H, m, ***^t^Bu***); 1.54 (2H, m, C***H_2_***(CH_2_)_2_COO); 1.61 (2H, m, CH_2_C***H_2_***CN); 2.29 (3H, s, C***H_3_***N); 2.33 (2H, t, J 7, C***H_2_***CNCH_3_); 2.74 (4H, m, NC***H_2_***C***H_2_***N); 2.64-2.95 (4H, dd, J 14, NC***H_2_***CN); 3.26 (4H, m, NC***H_2_***COO); 3.44 (2H, m, N(CH_3_)C***H_2_***COO), 3.66 (3H, s, OC***H_3_***). ^13^C NMR (CDCl_3_): δ 21.8 (***C***H_2_CH_2_COO); 25.9 (CH_2_***C***H_2_CN) 28.2 (C(***C***H_3_)_3_); 34.1 (***C***H_2_COOCH_3_); 36.9 (***C***H_2_CH_2_CN); 37.3 (***C***H_3_N); 51.4 (O***C***H_3_); 54.1 (N(CH_3_)***C***H_2_); 59.0 (N***C***H_2_***C***H_2_N); 62.2 (N***C***H_2_COO); 62.5 (N**C**H_2_CN); 62.8 (***C***N); 80.1 (N(CH_3_)CH_2_COO***C***); 80.8 (NCH_2_COO***C***); 164.2 (***C***OOCH_3_); 170.7 (N(CH_3_)CH_2_***C***); 172.4 (NCH_2_***C***). MS ES+ (m/z): found 586.4058 [M + H]^+^; C_30_H_56_N_3_O_8_ calcd for: 586.4067.

*5-[1,4-Bis-tert-butoxycarbonylmethyl-6-(tert-butoxycarbonylmethyl-methyl-amino)-[1,4]diazepan-6-yl]-pentanoic acid* **(5)**. ^1^H NMR (MeOD): δ 1.40 (4H, m, C***H_2_***C***H_2_***CN); 1.50-1.58 (27H, m, ***^t^Bu***); 1.64 (2H, m, C***H_2_***CH_2_COO); 2.37 (2H, t, J 7, CH_2_C***H_2_***COO); 2.84 (3H, s, C***H_3_***N); 2.97-3.05 (4H, m, NC***H_2_***C***H_2_***N); 3.24 (4H, dd, J 14, NC***H_2_***CN); 3.46 (4H, m, NC***H_2_***COO); 3.99 (2H, m, N(CH_3_)C***H_2_***COO). ^13^C NMR (MeOD): δ 17.3 (CH_2_***C***H_2_CN); 22.3 (***C***H_2_CH_2_COO); 25.1 (***C***H_2_CH_2_CN); 26.8 (2C, C(***C***H_3_)_3_); 27.0 (C(***C***H_3_)_3_); 32.8 (***C***H_2_COOH); 36.4 (***C***H_3_N); 51.7 (N(CH_3_)***C***H_2_); 53.8 (N**C**H_2_CN); 59.3 (N***C***H_2_***C***H_2_N); 59.2 (***C***N); 68.6 (N***C***H_2_COO); 81.5 (N(CH_3_)CH_2_COO***C***); 84.8 (NCH_2_COO***C***); 166.8 (N(CH_3_)CH_2_***C***); 169.9 (NCH_2_***C***); 175.8 (***C***OOH). MS ES^+^ (m/z): found 572.3911 [M + H]^+^; C_29_H_54_N_3_O_8_ calcd for: 572.3910.


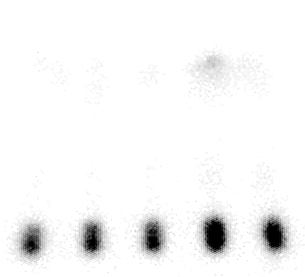


^68^Ga-DATATOC (Product)

Unchelated ^68^ Ga

Figure S1 RadioTLC for kit-type ^68^Ga-labelling of DATATOC at 1, 3, 5, 10 and 15 min.


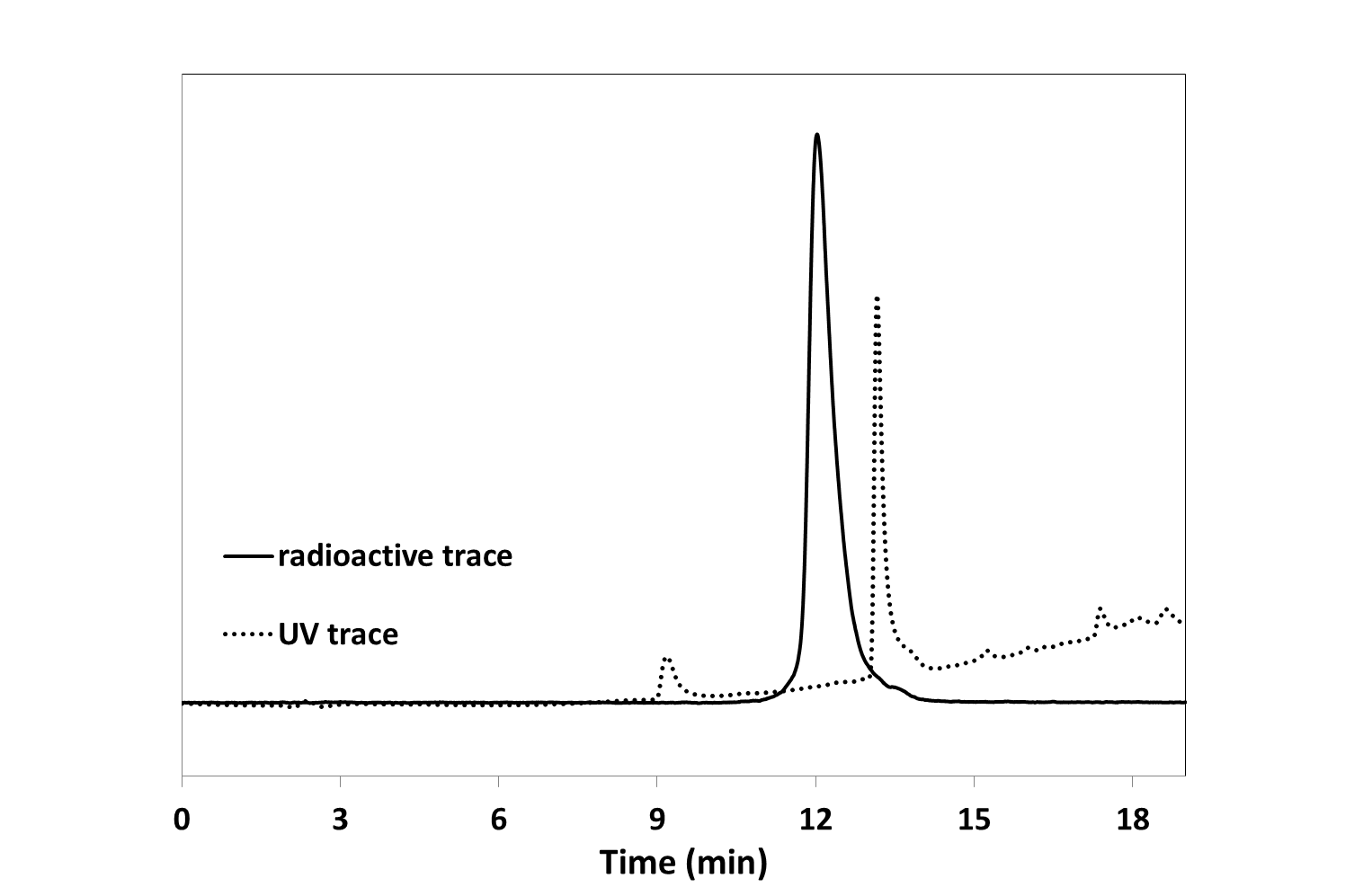


Figure S2 Radioactive (gamma) and UV traces for the kit-type labelling of DATATOC with ^68^Ga. The unlabelled ligand is evident at 9.11 min (confirmed by injection of the free ligand only) and the ^68^Ga-labelled complex at 12.97 min.


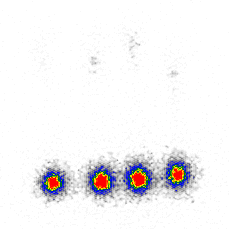


Figure S3 Illustrative example of RadioTLC for stability study of ^68^Ga-DATATOC 30, 60, 90 and 120 min after exposure to human serum.
